# A Bayesian approach to study the space time variation of leprosy in an endemic area of Tamil Nadu, South India

**DOI:** 10.1186/1476-072X-7-40

**Published:** 2008-07-21

**Authors:** Vasna Joshua, Mohan D Gupte, M Bhagavandas

**Affiliations:** 1 National Institute of Epidemiology, R127, Third Avenue, Tamil Nadu Housing Board Colony, Ayapakkam, Chennai 600 077, India; 2 Current address: Serum Institute of India Ltd, Hadapsar, Pune 411028, India; 3 TRP School of Management, SRM University, Chennai 603203, India

## Abstract

**Background:**

In leprosy endemic areas, patients are usually spatially clustered and not randomly distributed. Classical statistical techniques fail to address the problem of spatial clustering in the regression model. Bayesian method is one which allows itself to incorporate spatial dependence in the model. However little is explored in the field of leprosy. The Bayesian approach may improve our understanding about the variation of the disease prevalence of leprosy over space and time.

**Methods:**

Data from an endemic area of leprosy, covering 148 panchayats from two taluks in South India for four time points between January 1991 and March 2003 was used. Four Bayesian models, namely, space-cohort and space-period models with and without interactions were compared using the Deviance Information Criterion. Cohort effect, period effect over four time points and spatial effect (smoothed) were obtained using WinBUGS. The spatial or panchayat effect thus estimated was compared with the raw standardized morbidity (leprosy prevalence) rate (SMR) using a choropleth map. The possible factors that might have influenced the variations of prevalence of leprosy were explored.

**Results:**

Bayesian models with the interaction term were found to be the best fitted model. Leprosy prevalence was higher than average in the older cohorts. The last two cohorts 1987–1996 and 1992–2001 showed a notable decline in leprosy prevalence. Period effect over 4 time points varied from a high of 3.2% to a low of 1.8%. Spatial effect varied between 0.59 and 2. Twenty-six panchayats showed significantly higher prevalence of leprosy than the average when Bayesian method was used and it was 40 panchayats with the raw SMR.

**Conclusion:**

Reduction of prevalence of leprosy was 92% for persons born after 1996, which could be attributed to various intervention and treatment programmes like vaccine trial and MDT. The estimated period effects showed a gradual decline in the risk of leprosy which could be due to better nutrition, hygiene and increased awareness about the disease. Comparison of the maps of the relative risk using the Bayesian smoothing and the raw SMR showed the variation of the geographical distribution of the leprosy prevalence in the study area. Panchayat or spatial effects using Bayesian showed clustersing of leprosy cases towards the northeastern end of the study area which was overcrowded and population belonging to poor economic status.

## Background

Leprosy is a chronic infectious disease caused by the bacterium, *Mycobacterium leprae*, which can affect all ages and both sexes. Over the last two decades, prevalence of leprosy has come down substantially at the global level. Introduction and expansion of Multi drug therapy (MDT) in leprosy control programmes have dramatically lowered the prevalence level in almost all the endemic countries. For instance, in India leprosy prevalence has come down from 51 per 10,000 in 1981 to around 2.4 per 10,000 in March 2004 [1] and further below 1 per 10,000 by December 2005 [2].

In disease epidemiology, many of the infectious disease events do not occur randomly in geographical context but occur in clusters. In fact, leprosy epidemiology shows such uneven distribution between different geographic areas of the country, e.g. in China [3] and in Indonesia where in the leprosy endemic was high, the cases were extensively clustered and not equally distributed [4]. Hence, data analyses and interpretation should not ignore spatial dependence [5]. In India, we have observed that distribution of leprosy is uneven [6] even within the smallest community groups such as villages, right up to the family level [7].

Geographical or spatial analysis comes into play due to the existence of spatial dependence in data. Bayesian method lends itself for representing the spatial dependence during the estimation of model parameters. Application of Bayesian analysis in the field of leprosy is very limited [8]. A large controlled, double blind, randomized, prophylactic leprosy vaccine trial was conducted in South India to assess the prophylactic efficacies of four different candidate vaccines [9]. Within the trial area, we observed that leprosy was not randomly distributed and showed significant spatial dependence confirmed by using various measures of spatial autocorrelation like Moran's I, Geary's C and Kulldorff's SATSCAN statistics [10]. Hence, our main objective was to examine the variation in the prevalence of leprosy using four Bayesian models described by Arbyn etal [11] which was earlier proposed by Lagazio et al [12] and to explore possible factors that might have influenced these variations in the study area.

## Materials and Methods

Leprosy prevalence data from an endemic area, covering 148 panchayats (rural administrative units) comprising of 264 contiguous villages from Chingleput district, Tamil Nadu, South India was studied. This area was specifically identified for a leprosy vaccine trial [9] because of the high endemicity of leprosy. The entire population of about 300,000 people was screened by house to house examinations for leprosy and cases were identified at four time schedules between January 1991 and March 2003. Few socio- economic factors like population density and economic status were also collected.

### Definition of leprosy

A case of leprosy was defined as a person, having one or more of the following manifestations and who needed antileprosy treatment: hypopigmented or reddish skin lesion(s) with definite loss of sensation; damage to the peripheral nerves, as demonstrated by loss of sensation and or weakness of the muscles in parts supplied by these nerves; skin smear positive for acid fast bacilli.

The entire population in this area was screened for leprosy before vaccination. First, paramedical workers, trained in leprosy detection, screened the population. A proportion (5%) of this population was randomly allotted to "blinded" senior persons – either medical officers or senior paramedical officers for quality control. All cases and suspects detected by the junior paramedical workers were examined for diagnosis by two senior persons and by a third independent examiner in case of disagreement. Skin smear examination for detecting acid fast bacilli was done for all suspects and definite cases by the senior workers. A team of independent clinicians visited the field at frequent intervals to monitor the procedures for diagnosis of leprosy [9].

The data collected was also validated in many ways with the earlier surveys [7]. Hence the quality of data collected was remarkable and comparable to world standard as certified by the independent assessment committee consisting of national and international experts.

### Data analysis

Leprosy cases and population for each panchayat were cross-classified into 20 age groups (1–4,5–9,...,90–94,95–99) (there were no cases of leprosy under one year age) and four survey time periods (1991–93,1993–95,1997–98 &1999–2003). Since the time schedule for each of the survey was of varying length (January 1991 to March 2003) the mid-point of the four surveys (1992, 1994, 1997 & 2001) was considered for the time period. Cohorts were computed on the basis of survey time period and age. There were 20 overlapping or rolling birth cohorts like moving averages defined in this model, considering the mid-point, the cohorts were labeled as 1902, 1907,...,1997. The variation of prevalence of leprosy over space and time was modeled from January 1991 to March 2003 over 148 panchayats, after controlling for age. The term cohort effect refers to population born during a particular survey period identified by period of birth so that its characteristics can be ascertained as it enters successive and age strata [13].

Four Bayesian models, namely

(i) Space-Cohort (SC) with interactions,

(ii) Space-Cohort (SC) without interactions,

(iii) Space-Period (SP) with interactions and

(iv) Space-Period (SP) without interactions were fitted.

Models with and without interaction terms were compared using the Deviance Information Criterion (DIC) [14], being a generalization of the Akaike's Information Criterion in the Bayesian framework, i.e., lower the DIC values better the model. Posterior distributions (using the priors and the available data) of the parameters of interest were obtained using Gibbs sampling in WinBUGS [15]. The data for the SC model (observed and expected cases) were obtained by aggregating the data over the four time periods. The data for the SP model (observed and expected cases) were obtained by aggregating the data over the age groups. For the SC model the average of all the cohorts and for SP model the average of all periods was used as the reference.

The spatial effects were measured using each of the models. These smoothed effects were compared using the raw standardized morbidity rate (SMR) [unsmoothed].

We have dealt with subgroups like panchayats hence we used prevalence instead of incidence to get sufficiently larger number of cases to draw meaningful conclusions. Moreover if we use incidence rather than prevalence, we will have only three survey data leaving the baseline survey. Generally the second survey incidence (a mixed bag of old prevalence- missed and new cases) is not considered for vaccine efficacy and also for trend analysis. Hence we would be left with the third and fourth survey and ultimately this exercise of Bayesian model would not be possible to examine the trend over years. Hence as a pragmatic measure, we considered prevalence cases for this study.

In the study area majority of leprosy cases belonged to paucibacillary variety and the multibacillary cases constituted a smaller proportion (17.4%, 3.3%, 5.4% and 1.4% in the four surveys respectively). Fixed duration of MDT for six months was practiced all through the study period. For the purpose of analysis in this paper we restricted it only for paucibacillary variety. Hence, prevalence trends also indicate trends in leprosy incidence and both go hand-in-hand. Any change in beneficial effect that happens during the survey period decreases incidence as well as prevalence. Since we had limited data collected specifically by panchayat on socio-economic factors and there could be other important factors apart from the two mentioned above, we did not include any of these factors directly in the model. Though the period of study was very short, in the region, major remarkable changes have taken place both in the leprosy control programme and improvement in the socio-economic status. According to the World Development Indicators 2005 [16] the rural poverty in India declined from 53% to 27% between 1977–78 and 1999–2000 and the Indian social structure was transformed between 1991 and 2001 like increased literacy rate, urbanization, industrialization, new economic liberalization etc.

The formulae used are described in Appendix-1

## Results

There were 6,601 cases, 4,731 cases 3,342 cases and 2,098 cases of leprosy respectively at the four time periods. The DIC value for the space cohort model with and without interaction were compared. Since the models with the interaction term out- performed with smaller DIC values (Table 1 and Table 2). Further analyses were carried out with interaction term included into the SC and SP model. The Markov Chain Monte Carlo Simulation (MCMC) scalar parameters and their 95% credible intervals for the SC and SP models with interactions are shown in Table 3 and Table 4 respectively.

**Table 1:** Space-cohort model in two taluks of Tamil Nadu, South India.

	**Model without interactions**	**Model with interactions**
$\bar{D}$	12097.8	10605.9
$D\left(\bar{\theta }\right)$	11954.7	10474.6
DIC	12240.9	10737.2

**Table 2:** Space-period model in two taluks of Tamil Nadu, South India.

	**Model without interactions**	**Model with interactions**
$\bar{D}$	3880.5	3774.2
$D\left(\bar{\theta }\right)$	3757.4	3649.1
DIC	4003.7	3899.4

**Table 3:** Summary of Markov Chain Monte Carlo scalar parameters. Space Cohort Bayesian model with interactions.

**node**	**mean**	**sd**	**MC error**	**Median RR**	**95% CI**
***α***	0.004	0.056	0.004	0.011	-0.139, 0.096
***τ***^*struct*^	19.21	10.14	0.55	16.86	6.74,46.11
***τ***^*unstruct*^	24.49	8.87	0.44	22.70	14.29,46.33
***τ***^*cohort*^	4.53	1.53	0.02	4.35	2.06, 8.06
***τ***^*ac*^	5926	1746	131	6352	1651, 8460

**Table 4:** Summary of Markov Chain Monte Carlo scalar parameters. Space period Bayesian models with interactions.

**node**	**Mean**	**sd**	**MC error**	**Median RR**	**95% CI**
***α***	-0.136	0.029	0.001	-0.136	-0.194, -0.081
***τ***^*struct*^	26.45	19.11	1.01	21.35	6.78, 78.25
***τ***^*unstruct*^	25.38	28.45	1.61	21.46	13.91, 48.71
***τ***^*period*^	1490	1382	21	1084	125, 5282
***τ***^*ac*^	5563	810	35	5513	4128, 7274

### Cohort effect using the SC model with interaction

The median cohort effects declined from 1.86 to 0.08 over the successive cohorts. The cohort effect using SC model (Table 5) measured in terms of relative risk, was significantly higher than the average in the older cohorts C ≤ 11, i.e. persons born before 1957. The cohort effect steadily decreased up to 1922–1931 (C ≤ 6) and increased substantially during 1927–1936,1932–1941 (C = 7 & C = 8) and again significantly declined to a higher risk of 36% at C = 11 and finally reached to a lower risk of 92% (C = 20) than the average for persons born after 1996. There were four major jumps (difference between a cohort and the preceding one) observed in the risk pattern of the cohorts i.e. 1927–1936 (higher risk of 45%), 1942–51(reduced risk of 31%), 1987–1996 (reduced risk of 33%) and 1992–2001(reduced risk of 40%).

**Table 5:** Median Relative risk (cohort effects) and 95% credible intervals in two taluks of Tamil Nadu, South India.

**Rolling Cohort**	**Mid year**	**Index (C)**	**Median RR**	**95% CI**
-1906	1902	1	1.86	1.01, 4.22
1902–1911	1907	2	1.71	1.08, 2.98
1907–1916	1912	3	1.65	1.10, 2.41
1912–1921	1917	4	1.51	1.14, 1.97
1917–1926	1922	5	1.34	1.09, 1.63
1922–1931	1927	6	1.28	1.10, 1.49
1927–1936	1932	7	1.73	1.51, 1.96
1932–1941	1937	8	1.74	1.54, 1.96
1937–1946	1942	9	1.66	1.48, 1.87
1942–1951	1947	10	1.35	1.20, 1.51
1947–1956	1952	11	1.36	1.21, 1.52
**1952–1961**	**1957**	**12**	**0.99**	**0.88, 1.11**
1957–1966	1962	13	0.97	0.87, 1.09
1962–1971	1967	14	0.76	0.67, 0.85
1967–1976	1972	15	0.81	0.72, 0.91
1972–1981	1977	16	0.66	0.59, 0.74
1977–1986	1982	17	0.71	0.64, 0.80
1982–1991	1987	18	0.81	0.73, 0.91
1987–1996	1992	19	0.48	0.42, 0.55
1992–2001	1997	20	0.08	0.06, 0.10

### Period effect using the SP model with interaction

The period effect over four time points using SP model showed a significantly higher risk of 1.032 than the average, i.e. 3.2% to a lower risk 1.8% (Table 6).

**Table 6:** Median period effects and 95% credible intervals using Bayesian models in two taluks of Tamil Nadu, South India.

**Period**	**Median RR**	**95% CI**
April 1992	1.032	1.010, 1.070
May 1994	1.002	1.001, 1.026
November 1997	0.985	0.959, 1.007
March 2001	0.982	0.949, 1.013

### Space effect using SC and SP model with interaction

**Figure 1:**
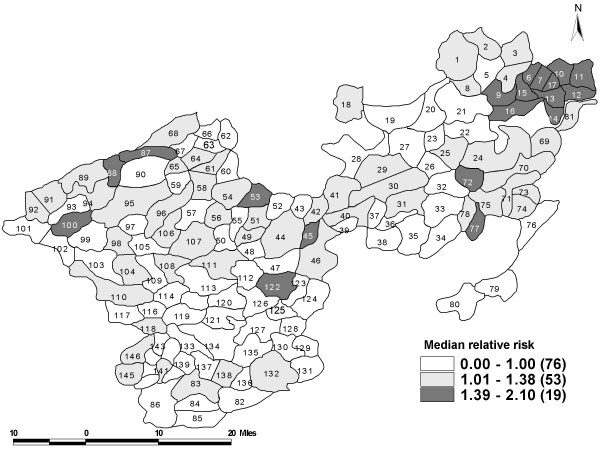
The median relative risk of leprosy prevalence across 148 panchayats estimated using the Bayesian model in two taluks of Tamil Nadu, South India (1991–2003).

**Figure 2:**
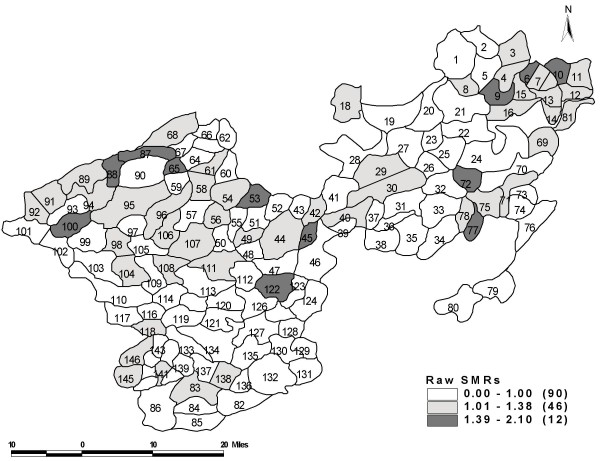
Geographical distribution of raw SMR across 148 panchayats in two taluks of Tamil Nadu, South India (1991–2003).

The spatial effect values (smoothed Bayesian) using SC and SP models using interaction terms were similar as observed in the Belgium study [11]. The spatial effects of different panchayats are listed in Table 7 and it can be further visualized in the choropleth map (Figure 1). The spatial effects varied between 0.59 and 2. Bayesian model identified 26 panchayats that had a significantly higher risk of leprosy. There was a higher risk of leprosy (50% or more) found in 10 panchayats and most of them lay close to each other towards the North-Eastern end of the study area. The lowest risk (relative risk 0.59) was observed in two panchayats. Raw SMR identified 43 panchayats at a higher risk, which was statistically significant (Figure 2); whereas it was 26 panchayats by the Bayesian models.

**Table 7:** Panchayat effect and 95% credible intervals of prevalence of leprosy in two taluks of Tamil Nadu, South India.

**Panch. No.**	**Median RR**	**95% CI**	**Panch. No.**	**Median RR**	**95% CI**
**072**	**2.00**	**1.64, 2.41**	140	1.00	0.85, 1.18
**006**	**1.88**	**1.71, 2.07**	142	0.98	0.80, 1.19
**010**	**1.73**	**1.46, 2.05**	050	0.97	0.75, 1.24
**009**	**1.66**	**1.48, 1.86**	043	0.97	0.85, 1.10
**015**	**1.55**	**1.35, 1.78**	105	0.97	0.77, 1.20
**012**	**1.54**	**1.38, 1.72**	055	0.95	0.72, 1.23
**017**	**1.54**	**1.37, 1.73**	057	0.95	0.76, 1.18
**077**	**1.54**	**1.23, 1.91**	085	0.95	0.75, 1.19
**088**	**1.51**	**1.23, 1.85**	021	0.95	0.77, 1.15
**045**	**1.50**	**1.20, 1.86**	005	0.95	0.81, 1.10
**053**	**1.49**	**1.26, 1.76**	137	0.94	0.83, 1.06
**014**	**1.48**	**1.26, 1.72**	099	0.94	0.77, 1.13
**013**	**1.47**	**1.27, 1.71**	135	0.93	0.76, 1.13
**087**	**1.47**	**1.25, 1.73**	119	0.93	0.76, 1.12
**016**	**1.44**	**1.29, 1.61**	066	0.92	0.69, 1.21
**100**	**1.44**	**1.18, 1.73**	027	0.92	0.73, 1.15
**007**	**1.43**	**1.25, 1.62**	102	0.92	0.75, 1.12
**122**	**1.42**	**1.18, 1.69**	123	0.92	0.70, 1.18
**011**	**1.41**	**1.21, 1.63**	097	0.92	0.65, 1.27
**138**	**1.38**	**1.25, 1.54**	033	0.91	0.74, 1.11
065	1.35	0.98, 1.86	134	0.90	0.76, 1.07
**091**	**1.34**	**1.09, 1.64**	039	0.89	0.63, 1.22
094	1.33	1.00, 1.60	038	0.89	0.70, 1.11
**075**	**1.32**	**1.15, 1.51**	067	0.89	0.67, 1.14
**081**	**1.32**	**1.14, 1.52**	062	0.88	0.70, 1.09
**145**	**1.29**	**1.16, 1.44**	144	0.87	0.73, 1.04
**069**	**1.29**	**1.13, 1.47**	133	0.87	0.70, 1.08
056	1.29	1.00, 1.56	052	0.87	0.68, 1.11
058	1.25	0.97, 1.59	048	0.86	0.63, 1.17
**030**	**1.24**	**1.12, 1.38**	115	0.86	0.64, 1.14
071	1.23	1.00, 1.47	136	0.86	0.68, 1.07
008	1.23	0.95, 1.57	084	0.85	0.69, 1.05
018	1.23	1.00, 1.48	131	0.84	0.67, 1.05
042	1.22	1.00, 1.40	037	0.84	0.68, 1.03
029	1.21	1.00, 1.34	034	0.84	0.67, 1.03
061	1.20	1.00, 1.43	022	0.83	0.65, 1.05
107	1.20	0.96, 1.49	120	0.83	0.63,1.07
049	1.18	0.93, 1.49	139	0.82	0.67, 1.00
092	1.18	1.00, 1.35	124	0.81	0.65, 1.01
004	1.18	1.00, 1.38	026	0.81	0.64, 1.01
003	1.17	1.00, 1.36	093	0.80	0.60, 1.05
078	1.17	0.91, 1.47	023	0.80	0.60, 1.05
089	1.16	0.99, 1.36	060	0.80	0.58, 1.09
096	1.16	0.91, 1.45	082	0.80	0.64, 0.98
070	1.15	1.01, 1.31	147	0.80	0.64, 0.98
098	1.15	0.91, 1.44	148	0.79	0.62, 1.00
146	1.14	0.99, 1.30	032	0.79	0.63, 0.99
054	1.14	0.94, 1.36	127	0.79	0.62, 1.00
025	1.12	0.91, 1.38	129	0.78	0.60, 1.01
118	1.12	0.91, 1.36	080	0.78	0.64, 0.95
111	1.12	0.90, 1.37	116	0.77	0.58, 1.01
141	1.12	0.86, 1.43	035	0.77	0.59, 0.98
040	1.11	0.88, 1.39	020	0.76	0.61, 0.94
002	1.11	0.90, 1.35	019	0.75	0.59, 0.95
068	1.11	0.94, 1.31	126	0.75	0.62, 0.89
106	1.10	0.82, 1.46	112	0.74	0.55, 0.97
044	1.09	0.94, 1.26	117	0.73	0.62, 0.85
024	1.09	0.90, 1.31	114	0.73	0.55, 0.96
083	1.08	0.93, 1.25	128	0.70	0.57, 0.86
001	1.07	0.92, 1.24	130	0.70	0.54, 0.89
073	1.07	0.87, 1.30	121	0.70	0.53, 0.90
095	1.07	0.88, 1.28	047	0.70	0.55, 0.87
051	1.06	0.85, 1.31	113	0.69	0.54, 0.88
031	1.06	0.87, 1.29	109	0.69	0.54, 0.87
074	1.06	0.91, 1.22	059	0.67	0.52, 0.86
108	1.05	0.85,1.29	103	0.64	0.52, 0.79
104	1.04	0.83, 1.30	090	0.64	0.49, 0.83
046	1.04	0.87, 1.24	125	0.64	0.50, 0.81
110	1.04	0.89, 1.20	086	0.63	0.51, 0.76
041	1.04	0.83, 1.30	063	0.62	0.46, 0.82
132	1.03	0.91, 1.17	079	0.62	0.51, 0.74
064	1.02	0.82, 1.26	076	0.60	0.46, 0.77
101	1.00	0.80, 1.25	143	0.59	0.47, 0.74
028	1.00	0.83, 1.21	036	0.59	0.39, 0.85

Panchayats are arranged in descending order of median RR values for sake of comparison. Significant panchayats and RR greater than one are highlighted.

## Discussion

We examined variation in prevalence of leprosy using Bayesian methods over 20 rolling cohorts and four time periods from a meticulously collected dataset of a vaccine trial conducted in South India. Observing the cohort effects it neither showed a steady decreasing nor an increasing effect. It showed an intricate effect, whereas leprosy prevalence trends in period effects has decreased continuously over four time points.

The steady decreasing period effect reflects that the control programme had reached all the target population whereas the intricate cohort effects showed that the effects captured were not similar in all the cohorts.

The higher difference in the risk in successive **cohorts **could be attributable to persons coming forward for seeking treatment, gradual awareness in the community that leprosy is curable, slowly combating the social stigma, early screening programmes, better case detection methods, availability of therapies and elimination programmes. This is particularly true in the vaccine trial setting where health systems operations and leprosy programme have been implemented in total [6].

The turning point in the risk pattern (from high risk to low risk) occurred during the cohort 1952–1961 (C = 12). This change could be due to the impact of Dapsone based National Leprosy Control Programme introduced during 1955. Gradual reduction of risk in the latter cohorts (C = 13, C = 14) may be due to continuing effect of programs and possibly improvement in socio-economic conditions the transition that has been taking place in India. Further reduction in risk (C = 19) could be due to more effective and intensified treatment programmes like Multi-Drug Therapy in 1991, that has changed the face of leprosy [17] and introduction of effective prophylactic vaccines through the leprosy vaccine trial [9].

Our data indicate 92% reduction in the leprosy prevalence for persons born after 1996 (C = 20). The case detection and treatment activity in the community has brought down the new infection rate in the younger age group as a secondary effect of MDT in addition to the primary effect of prophylactic vaccines. The older age group might have been infected in the past i.e. before the introduction of Dapsone and MDT as well as before the introduction of the vaccination programme. They might already be harbouring the infection and the break down could result in fresh disease. This is similar to the endogenous reactivation observed in tuberculosis [18].

We observed slow decline in the estimated **period effects **of leprosy in the study area. It agrees with the fact that the prevalence of leprosy has come down over years globally as well as in India [19]. Leprosy being a chronic disease, temporal changes within endemic regions are slow [7]. This phenomenon is observed in the study area. Moreover, the decline in the risk of leprosy over the time periods in the study area could be due to better understanding on nutrition, hygiene, and increased public understanding of the disease (socio-economic factors) which limit the spread of leprosy or increased resistance to leprosy.

The **spatial effect **using Bayesian model was compared with the raw SMR gives the variation in the geographical distribution of the leprosy prevalence. The use of Bayesian smoothing approach accounts for the variability in the population at risk and clustering effect. Observing the spatial Bayesian effect, there was a strong pattern of clustering towards the North-Eastern region of the study area. Though the effects obtained through the models showed the reduction in the risk of prevalence of leprosy still a few pockets of high prevalence exist. The situation was similar in Tuscany [11] where the epidemic of lung cancer when analyzed using birth cohorts showed a decline but the spatial pattern was evident and strong towards the north-west/south-east gradient.

In the state of Ceara North- East Brazil [20], the spatial pattern of the leprosy disease was heterogeneous and municipalities with very high prevalence were clustered towards the North-South axis. Surprisingly the region with the highest incidences were most urbanized and economically developed. According to the authors the reasons for spatial clustering of disease rates might be related to a heterogeneous distribution of the factors such as crowding, social inequality and environmental characteristics which by themselves determine the transmission of Mycobacterium leprae. It could also be due to more efficient health system present in these regions to detect new cases of leprosy more efficiently. We tried to explore the authors' above perception and observed that the pockets identified by the Bayesian model had a population density of about 1,427 per sq km which is two times higher than the district and three times higher than the state population density [21]. Hence, possibly as result of this, environmental factors, such as urbanization and overcrowding due to inadequate housing, could have led to more frequent close contact with the source of infection and favoured the spread of leprosy. We also observed that nearly 37% of the people from this pocket belong to the economically poorer strata. Generally people from economically poorer strata are more prone to infectious diseases like leprosy as they live in close proximity to one another resulting in higher risk of contracting the disease [22]. Dharmendra [23] emphasizes that one cannot control, eliminate or eradicate leprosy without improving the socio-economic status or changing the in sanitary habits of the common people. Hence these few pockets or strata need greater care to bring down the leprosy prevalence to much greater extent and to make the study area free from leprosy.

## Appendix-1

The step by step algorithms and winbug codes can be downloaded from annexes of Arbyn etal [11].

Let *O*_*ijt *_denote the observed count of leprosy cases in panchayat i (i = 1, 2,...,148) in the j^th ^cohort (j = 1, 2 ..... 20) and during the time point t (t = 1, 2, 3 & 4);

$O_{ij}=\sum_{t=1}^{4}O_{ijt}$ be the observed number of leprosy cases in the i^th ^panchayat and j^th ^cohort;

${\text{X}}_{ij}=\sum_{t=1}^{4}{\text{X}}_{ijt}$ be the expected number of leprosy cases in the i^th ^panchayat and j^th ^cohort;

*O*_*ij *_~ Poisson (*α*_*ij*_),

with *α*_*ij *_= *rr*_*ij *_. X_*ij*_, where i = 1, 2,...,148 panchayats,

j = 1, 2 ..... 20 cohorts,

$$\left(\log\left(\frac{\alpha_{ij}}{X_{ij}}\right)\right)=\log\left(rr_{ij}\right)=\xi_{ij},$$

where

*ξ*_*ij *_is a linear predictor,

*rr*_*ij *_is the relative risk of the i^th ^panchayat and the j^th ^cohort.

$rr_{ij}=e^{\xi_{ij}}$ is the estimated cohort effects and similar to the standardized cohort morbidity ratios described by Beral [24].

*ξ*_ij_, the linear predictor can be specified in two ways.

Model without interactions: *ξ*_*ij *_= a + *β*_i_^str ^+ *β*_i_^unstr ^+ *β*_j_^coh^,

Model with interactions: *ξ*_ij _= a + *β*_i_^str ^+ *β*_i_^unstr ^+ *β*_j_^coh ^+ SS_ij_^ac^,

Where SS_ij_^ac ^= S_ij_^ac ^- S_1j_^ac ^- S_i1_^ac ^+ S_11_^ac ^and

The first term S_ij_^ac ^represents the interaction term of panchayat and cohort and SS_ij_^ac ^is the centering used to improve a convergence of the Markov Chain Monte Carlo Simulation (MCMC) same as discussed in detail by Arbyn et al [11].

Where *β*_i_^str ^represents structured spatial variability;

*β*_i_^unstr ^represents unstructured spatial variability;

*β*_j_^coh ^represents the effect of the j^th ^cohort;

Prior distribution for the model

*β*^str ^= (*β*_1_^str^,......, *β*_148_^str^)^T^,

*β*^unstr ^= (*β*_1_^unstr^,......, *β*_148_^unstr^)^T^,

*β*^coh ^= (*β*_1_^coh^,......, *β*_20_^coh^)^T^,

S^ac ^= (S^ac^_1,1_,......, S^ac^_148,20_)^T^.

The priors are multivariate normals.

*β*^str^~*N*(0,(*μ*_*str*_*σ*_*str*_)^-1^),

*β*^unstr^~*N*(0,(*μ*_*unstr*_*I*_148_)^-1^),

*β*^coh^~*N*(0,(*μ*_*coh*_*σ*_*coh*_)^-1^),

S^ac^~*N*(0,(*μ*_*ac*_*σ*_*ac*_)^-1^).

The structured spatial term *β*^str ^and cohort effect *β*^coh ^are assigned the Gaussian Conditional Autoregression (CAR) prior distribution. They followed the closer specification of matrices *σ*_*str*_, *σ*_*coh *_and *σ*_*ac *_as mentioned by Lagazio [12]. They are implemented using WinBUGS' function car.normal().

The above function constraints, the random effects to add up to zero, so that the following constraints are satisfied in the model.

$$\begin{array}{cc} \sum_{i=1}^{148}\beta_{i}^{str}=0, & \sum_{j=1}^{20}\beta_{j}^{coh}=0. \end{array}$$

The prior for the interaction vector S^ac ^is a Markov random field.

The intercept term 'a' was given a flat prior through WinBUGS function dflat().

The precision terms *μ*_*str *_*and μ*_*coh *_and *μ*_*unstr *_were given Gamma priors.

The interaction precision parameter *μ*^ac ^discussed in detail [11] was also given the gamma prior closely to Lagazio [12].

The space period model is similar as above instead of cohorts, periods used (t = 4). The algorithmic steps for the four models using WINBUGS with and without interactions similar to the space cohort model are discussed in detail elsewhere [11].

Deviance Information Criterion (DIC) is defined as

$DIC=\bar{D}+\left(\bar{D}-D\left(\bar{\theta }\right)\right)$ where

$\bar{D}$ – the posterior expectation of the deviance and summarizes the fit of the model,

$D\left(\bar{\theta }\right)$ – the deviance evaluated at the posterior expectations of parameters.

$\left[\bar{D}-D(\bar{\theta })\right]$ – the effective number of parameters.

In the model the spatial effect (autocorrelation) depends on

(i) whether any two panchayats share a common boundary and

(ii) the number of shared neighbours (panchayats).

For fitting the models with and without interactions, two chains, with 1:10 thinning was used to obtain a sample of 10 000 values.

In case of the model without interactions a burn-in of 50,000 iterations and an additional 50,000 iterations were used. For fitting the models with interactions, a burn-in of 100 000 iterations and an additional 50,000 were used.

Convergence was checked using procedures mentioned by Gelman and Geweke [25,26] test and partial correlation plots to check for achieved convergence, of relative risks and hyper-parameters.

## Competing interests

The authors declare that they have no competing interests.

## Authors' contributions

VJ conceived the ideas, performed the statistical analysis and drafted the manuscript. MDG conceived the study, coordinated and participated in the trial, gave critical and intellectual comments for the improvement of the manuscript. MB contributed to data analysis and gave critical comments for the improvement of the manuscript. All the authors read and approved the final manuscript.
